# Cross-modal and multisensory training may distinctively shape restored senses

**DOI:** 10.3389/fnins.2014.00450

**Published:** 2015-01-12

**Authors:** Jean-Paul Noel, Antonia Thelen

**Affiliations:** ^1^Neuroscience Graduate Program, Vanderbilt University Medical CenterNashville, TN, USA; ^2^Vanderbilt University Medical Center, Vanderbilt Brain Institute, Vanderbilt UniversityNashville, TN, USA

**Keywords:** multisensory, cross-modal, cochlear implant, spatial localization

The coupling of recent technological advances and conceptual understandings within the field of systems neuroscience, and in particular in the study of cross- and multisensory systems, has given rise to the development of a host of sensory substitution and restorative devices. These either leverage a particular sensory modality in order to compensate for loss in another or at least partly rely on a secondary sensory system in order to compensate for missing information. It is under this context that the study of cross-modal (i.e., transfer between sensory modalities) and multisensory (i.e., integration across different sensory modalities) training paradigms has provided information of vital importance (see Sharma et al., 2014). Isaiah et al.s' (2014) contribution in *the Journal of Neuroscience* describing the impact of audio-visual training on auditory localization in ferrets with cochlear implants (CIs) is one of the most recent examples of these efforts.

Ferrets were deafened either around the onset of hearing or as adults and submitted to either unilateral or bilateral cochlear implantation (UniCI and BiCI, respectively). Following a period of auditory and/or interleaved auditory and visual localization training, approach-to-target accuracy and head orienting responses were examined. In addition, various aspects of neuronal response in primary auditory cortex (A1) were measured as a function of time of hearing loss onset (early vs. late) and sensory training (none, auditory, or audio-visual).

Behaviorally, animals in the UniCI group were unable to localize auditory stimuli regardless of the duration of deafness and training provided. In contrast, late-onset hearing loss BiCI animals performed significantly above chance after auditory training, both in terms of approach-to-target behavior and initial head-orienting responses. Early-deafened BiCI ferrets could not localize sounds beyond chance, and unisensory auditory training did not improve target localization even after repeated sessions. Subsequently, these animals (both UniCI and BiCI) were trained on an interleaved auditory and visual paradigm in an attempt to achieve more accurate auditory localization. After cross-modal training early-deafened BiCI ferrets' auditory localization improved significantly. Importantly, this facilitation was sustained in ensuing unisensory auditory-only localization sessions.

Electrophysiological findings suggested that the behavioral improvements were likely a consequence of increased responsiveness and selectivity of neurons in A1. After interleaved visual and auditory training, neurons in ferret's A1 responded more vigorously and selectively to stimulation provided by the CI. This suggests a putative mechanism underpinning the behavioral improvements. However, the work also raises a number of interesting questions.

First, Isaiah et al. (2014) did not directly investigate the impact of a “classic” multisensory training paradigm (one in which the auditory and visual stimuli would be aligned in space and in time), but rather employed a training paradigm in which information was provided in an interleaved fashion. This raises an interesting question with regard to the brain circuits mediating the changes in A1 responsiveness and the associated behavioral benefits. Are these changes driven by activation differences in multisensory areas (e.g., temporal/parietal) or in reward-related regions (e.g., prefrontal)? In fact, prior research has repeatedly shown that multisensory training can improve unisensory performance through engagement of a wide spread cerebral network (Cappe et al., 2009; Shams and Kim, 2010). Furthermore, Isaiah et al. (2014) findings are in line with a model where cross-modal transfer is mediated by frontal areas since audition and vision were never conjointly activated, and therefore there is no reason to postulate that multisensory areas alone serve as a fundamental node in the computation leading to a facilitated auditory localization (for a review see Ettlinger and Wilson, 1990). Indeed, the authors propose that perhaps it is the prefrontal cortices that are driving cross-modal localization training and the enhanced responsiveness and selectivity exhibited by A1.

Similarly, human psychophysical and neuroimaging literature has repeatedly shown remapping effects to occur for auditory spatial representations after both cross-modal and multisensory training (for a review see Chen and Vroomen, 2013). The spatial ventriloquist aftereffects (Radeau and Bertelson, 1977) are a behavioral example of such an auditory spatial remapping due to vision. Further, evidence from human neuroimaging studies suggests the contribution of a fronto-temporo-parietal network in cross-modal and multisensory spatial cognition (for a review see Koelewijn et al., 2010). Nonetheless, a more mechanistic understanding of how this network comes to modulate A1 responsiveness and selectivity after interleaved visual trials and in the lack of spatiotemporal congruency remains unanswered. When framed from the perspective of sensory substitution devices, the overarching question for these experiments is whether genuine cross-modal plasticity occurred in multisensory networks, or whether reward networks mediated the perceptual learning?

Sensory loss leads to extensive cross-modal plasticity (Bavelier et al., 2006). In the case of congenitally deaf individuals, for instance, neural substrates in the auditory cortex might be recruited by other sensory modalities. Finney and MDobkins (2001) showed responses to visual motion in auditory cortex of deaf individuals. In addition, this plasticity seems to be the basis for the behavioral benefit auditory deprived individuals show in processing visual motion in the peripheral visual field (Bavelier et al., 2006). On the other hand, cross-modal reorganization of the deprived cortex can also be deleterious. By supporting processes grounded in another sensory modality, cross-modal plasticity might hinder cortical recruitment by the native sensory system. That is, electrical input to the auditory cortex after cochlear implantation might be inefficient if the cortical structure has been functionally reorganized by the spared sensory modalities. Accordingly, Lee et al. (2001), reported that deaf individuals in whom cross-modal plasticity was the most extensive were the least likely to benefit from CIs. An open question is whether a training paradigm based on invoking changes in prefrontal networks such as the cross-modal approaches employed here would be more or less effective than approaches founded on invoking changes in multisensory cortical networks derived from direct multisensory training methods.

The question becomes, could cross-modal training have long-term detrimental effects, as well as the short-term beneficial effects Isaiah et al. (2014) demonstrate? A key issue remains whether one type of training (e.g., cross-modal) would incite cortical plasticity more readily than the other (e.g., multisensory), and even whether the nature of this putative neuroplasticity would be akin in both conditions? Likely cross-modal and multisensory training will both result in cortical changes—the nature of which could be very different and which may be used in different ways when thinking about sensory substitution and restoration.

## Conflict of interest statement

The authors declare that the research was conducted in the absence of any commercial or financial relationships that could be construed as a potential conflict of interest.
